# Clinical Features and Treatments of Syphilitic Uveitis: A Systematic Review and Meta-Analysis

**DOI:** 10.1155/2017/6594849

**Published:** 2017-06-29

**Authors:** Ting Zhang, Ying Zhu, Gezhi Xu

**Affiliations:** ^1^Department of Ophthalmology, Eye and Ear Nose Throat Hospital, Shanghai Medical School, Fudan University, 83 Fenyang Road, Shanghai 200031, China; ^2^Department of Ophthalmology, Xiangya Hospital, Central South University, 87 Xiang Ya Road, Changsha 410008, China; ^3^Key Laboratory of Visual Impairment and Restoration, 83 Fenyang Road, Shanghai 200031, China

## Abstract

**Purpose:**

To investigate the clinical features and efficacies of treatments for syphilitic uveitis.

**Methods:**

PubMed was searched for studies of syphilitic uveitis published between January 1990 and October 2016. The clinical features were summarized and appraised. The pooled success rate was defined as an improved or maintained final visual acuity and was calculated with 95% confidence intervals (CIs). Heterogeneity, subgroup analysis, sensitivity analysis, and publication bias were assessed.

**Results:**

Thirty-two studies involving 670 patients were analyzed. The most common type of syphilitic uveitis was papillitis. The pooled success rate was 0.91 (95% CI 0.84–0.97) for antibacterial agents alone (15 studies, 286 patients); 0.95 (95% CI 0.91–0.98) for antibacterial agents and systemic corticosteroids combined (11 studies, 245 patients); and 0.91 (95% CI 0.80–0.98) for antibacterial agents, systemic corticosteroids, and other immunosuppressants combined (3 studies, 73 patients). Subgroup analyses revealed no correlations of the efficacy of antibacterial agent monotherapy with study characteristics, such as human immunodeficiency virus coinfection status.

**Conclusions:**

This systematic review and meta-analysis revealed the efficacy of antibacterial agents for treating syphilitic uveitis. Coadministration of systemic corticosteroids or immunosuppressants did not elicit further improvements in the clinical outcomes of antibacterial agents.

## 1. Introduction

Syphilis is a sexually transmitted chronic disease caused by the spirochete *Treponema pallidum,* which can be spread via mother-to-child transmission (congenital syphilis) or acquired in adulthood (acquired syphilis). It was estimated that there were 5.6 million new cases of syphilis worldwide in 2012, with a global prevalence of 0.5% among people aged 15–49 years [1]. According to a 22-year survey of incident uveitis cases, the actual frequency of syphilitic uveitis was <1%, but the incidence has risen markedly [2].

Syphilitic uveitis is an infectious type of uveitis that should be included in the differential diagnosis of any form of ocular inflammation. Syphilitic uveitis can occur at any stage of acquired syphilis. It can result in visual loss if it is unrecognized or if it is mistreated as a noninfectious ocular inflammation. It can affect various parts of the eye and may present as anterior uveitis, posterior uveitis, panuveitis, retinitis, papillitis, and even scleritis, making it a “great masquerader” [3]. Misdiagnosis could lead to unnecessary or even harmful therapies, resulting in deterioration of uveitis and possibly even the patient's general health. However, syphilitic uveitis is curable with early aggressive use of antibacterial agents, making its prompt diagnosis a clinical necessity.

To manage syphilis, European (International Union against Sexually Transmitted Infections) [4] and United States (Centers for Disease Control and Prevention (CDC)) [5] guidelines recommend standard use of intravenous benzyl penicillin at a dose of 12–24 million units per day, with 3-4 million units given every 4 h, for 10–21 days. The recent World Health Organization Sexually Transmitted Infection (STI) guidelines recommend benzathine penicillin G administered intramuscularly at a dose of 2.4 million units once weekly for three consecutive weeks to treat late syphilis (including ocular syphilis) [6].

Because immunological reactions are also believed to be involved in the pathogenesis of late syphilis [7], it seems reasonable to administer corticosteroids or other immunosuppressants in combination with standard antibacterial regimens to treat syphilitic uveitis. However, there is limited evidence regarding their efficacy of corticosteroids or other immunosuppressants for treating syphilitic uveitis.

Syphilis and human immunodeficiency virus (HIV) coinfection is common [8], and the prevalence of ocular syphilis in HIV-positive patients was very high, 9%, in an earlier study [9]. The immune status of these patients might be complicated, resulting in alterations of the clinical or laboratory manifestations of syphilis, increased risk of syphilitic complications, and diminished responses to antibacterial agents. The infection was also common in posterior regions of the eye in patients coinfected with HIV in several studies [9, 10]. Considering these issues, the outcomes of antibacterial agents in patients with syphilitic uveitis and HIV are of particular interest.

The objectives of this systematic review and meta-analysis were to summarize the clinical and laboratory features of patients with syphilitic uveitis and to assess the efficacy of antibacterial agents administered alone or in combination with other agents.

## 2. Methods

### 2.1. Electronic Database Searches

PubMed was searched in October 2016 using the terms “ocular [All Fields],” “intraocular [All Fields],” or “uveitis [All Fields],” which were then matched with “syphilis [All Fields]” or “syphilitic [All Fields].” Articles published between January 1990 and October 2016 were retrieved, and the reference lists of the retrieved articles were manually checked for relevant articles. We also searched the Web of Science for articles citing the articles retrieved from PubMed, and the additional articles were assessed for possible inclusion. Observational studies (including retrospective and prospective cohort studies, case-control studies, cross-sectional studies, case series, and clinical studies) that reported the clinical features of syphilitic uveitis and their treatment outcome were included. The exclusion criteria were as follows: studies that did not report the full syphilitic uveitis spectrum or did not focus on the clinical features of syphilitic uveitis; studies with <10 patients; articles published before 1990; and non-English-language studies.

### 2.2. Data Collection and Analysis

Two independent authors (TZ and YZ) conducted the electronic and manual searches using the predetermined inclusion and exclusion criteria, and the full text of all potentially eligible studies were assessed. Any differences in study selection between the two authors were referred to a third author (GZX) and were resolved by discussion. The data were extracted from all articles by one author (TZ) and were verified by a second author (YZ). Data were collected using pre-prepared forms covering study design, patient demographics, clinical presentation, diagnosis, interventions, treatment outcomes, and factors associated with treatment outcomes.

### 2.3. Assessment of Risk of Bias

Two authors (TZ and YZ) independently assessed the risk of bias using the Newcastle-Ottawa Quality Assessment Scale (NOS). The NOS comprises three domains: selection representativeness (four items), comparability (two items), and ascertainment of either the exposure or outcome (three items). Each item was given one star if addressed. Scores of >6 stars, 6 stars, and <6 stars were considered to represent low risk of bias, medium risk of bias, and high risk of bias, respectively. Any discrepancy in the assessment of bias was resolved by discussion.

### 2.4. Data Synthesis

The meta-analysis was performed in accordance with PRISMA guidelines [11]. The pooled success rate was defined as the percentage of eyes with improved or maintained final visual acuity after treatment. The pooled success rates with 95% confidence intervals (CI) were compared between treatment modalities using a random-effects model (DerSimonian-Laird method) or a fixed-effects model (Mantel-Haenszel method). A *P* value of <0.05 was used to indicate statistical significance. Cochran *Q* and *I*^2^ tests were also performed to investigate study heterogeneity [12]. An *I*^2^ statistic of <50% was regarded as low heterogeneity and an *I*^2^ statistic of >75% was regarded as substantial heterogeneity. Forest plots were used to display the effects of different treatments. Funnel plots with Egger's and Begg's regression were also drawn to detect possible publication bias [13]. Subgroup analyses were also done to identify the correlation of the efficacy of antibacterial agents alone with relevant study characteristics (year of publication, number of involved eyes, HIV coinfection status, follow-up duration, and the geographical location of patients). R software (version 3.3.1) with the meta package was used for the meta-analyses.

## 3. Results

### 3.1. Studies Analyzed

**Figure 1:**
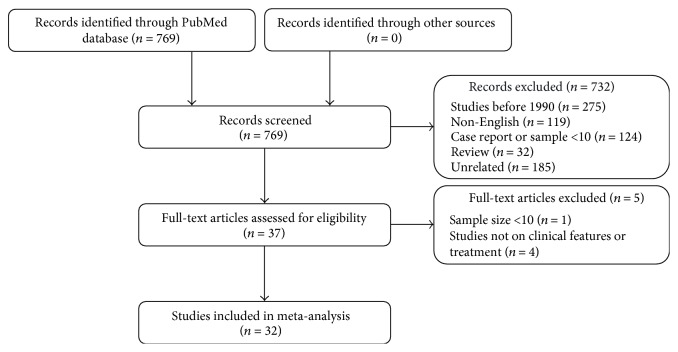
Flow chart of the study selection process.

A total of 769 articles were initially retrieved from PubMed (Figure 1), of which 32 were considered eligible after full-text review and were included in our analysis [14–45]. Most studies (31/32) were retrospective; only one was a prospective study [26]. Nine studies compared the clinical features and treatment outcomes between patients coinfected with HIV versus patients without HIV infection [17, 19, 22, 23, 26, 27, 33, 34, 37].

Using the NOS, 17 studies had a score of 5 stars, 11 studies had a score of 4 stars, 3 studies had a score of 3 stars, and 1 study had a score of 1 star.

### 3.2. Patient Demographics

The included studies comprised 670 patients, and the number of eyes in each study ranged from 11 to 139 eyes (Table 1). The mean or median age of patients ranged from 37 to 58 years. Thirty studies reported a male preponderance, and the cumulative mean proportion of men was 77.5%. Nineteen studies reported sexual orientation, and the cumulative mean proportion of men who have sex with men was 50% in these studies.

### 3.3. Clinical Features and Diagnostic Criteria of Syphilitic Uveitis

Sixteen studies reported the presence of systemic syphilis with a cumulative percentage of 38.5%. Bilateral involvement was more common than unilateral involvement (cumulative mean 62.1%; bilateral:unilateral ratio 1.6 : 1) in the studies that reported this information (31/32 studies) (Table 2). Twenty-eight studies reported syphilitic uveitis with HIV coinfection, but the percentage of HIV coinfected patients ranged considerably from 7.7% to 100%. Seven studies reported whether HIV infection was newly diagnosed after the diagnosis of syphilis, and the percentage of affected patients ranged from 16.7% to 63.6%.

The diagnosis of syphilitic uveitis in all studies was based on clinical features (symptoms, signs, and axillary examinations) together with laboratory test confirmation. Serologic nontreponemal (rapid plasma reagin test) and treponemal specific tests (fluorescent treponemal antibody absorption test) were the most commonly used tests, being used in 17 and 21 studies, respectively. Other confirmatory laboratory tests included *Treponema pallidum* particle agglutination assays in nine studies and *Treponema pallidum* hemagglutination assays in eight studies. Twenty-nine studies also performed treponemal and reagin tests using cerebrospinal fluid (CSF) samples for the diagnosis of neurosyphilis. The venereal disease research laboratory test (VDRL) was the most common test used to detect neurosyphilis (22/29) with a cumulative positivity rate of 34.8%. CSF white blood cell (WBC) counts were reported in 9 studies, and the median WBC count ranged from 2 to 11 cells/*μ*l. CSF protein levels were reported in 8 studies, and the median protein level ranged from 42 to 464 mg/dl.

The clinical classification of uveitis was reported using the standardization of uveitis nomenclature (SUN) [46] in 10 studies and the standard of International Uveitis Study Group (IUSG) [47] in 4 studies and was not specified in 18 studies. No studies included local investigations, such as culture or polymerase chain reaction of intraocular fluid, for the diagnosis of syphilitic uveitis. Four studies reported the response to antibacterial treatment as part of the diagnosis of syphilitic uveitis.

We extrapolated further information and summarized the results according to the standardization of uveitis nomenclature, which comprises the following: anterior uveitis, intermediate uveitis, posterior uveitis, panuveitis, retinitis and retinal vasculitis, necrotizing retinitis, choroiditis, serous retinal detachment, macular edema, neuroretinitis, papillitis, and optic edema. Other phenotypes such as acute syphilitic posterior placoid choroiditis and increased intraocular pressure were also assessed. Our review revealed that the optic disc (reported in 28 studies) was the most frequently affected site (presented as papillitis, optic neuritis, or neuroretinis), and 24 studies reported syphilitic uveitis as panuveitis (Table 3).

### 3.4. Management of Syphilitic Uveitis and Clinical Outcomes

The drugs used, regimens, route of administration, treatment duration, and follow-up duration varied considerably (Table 4). Intravenous penicillin was used in 30 studies. Ceftriaxone and macrolide antibacterial agents (e.g., doxycycline or tetracycline) were used in 12 and 9 studies, respectively, in case of penicillin allergy. Additional systemic corticosteroids were used in 14 studies with a cumulative mean of 43.8% of patients. Immunosuppressants were used in 3 studies (cumulative mean of 9.4% of patients) and included methotrexate, mycophenolate mofetil, cyclophosphamide, and cyclosporine. Therefore, the patients included in this review received the following regimens: antibacterial agents alone (antibacterial monotherapy); antibacterial agents and systemic corticosteroids (double therapy); or antibacterial agents, systemic corticosteroids, and immunosuppressants (triple therapy). The mean follow-up time ranged from 2.1 to 35 months.

### 3.5. Antibacterial Monotherapy

**Figure 2:**
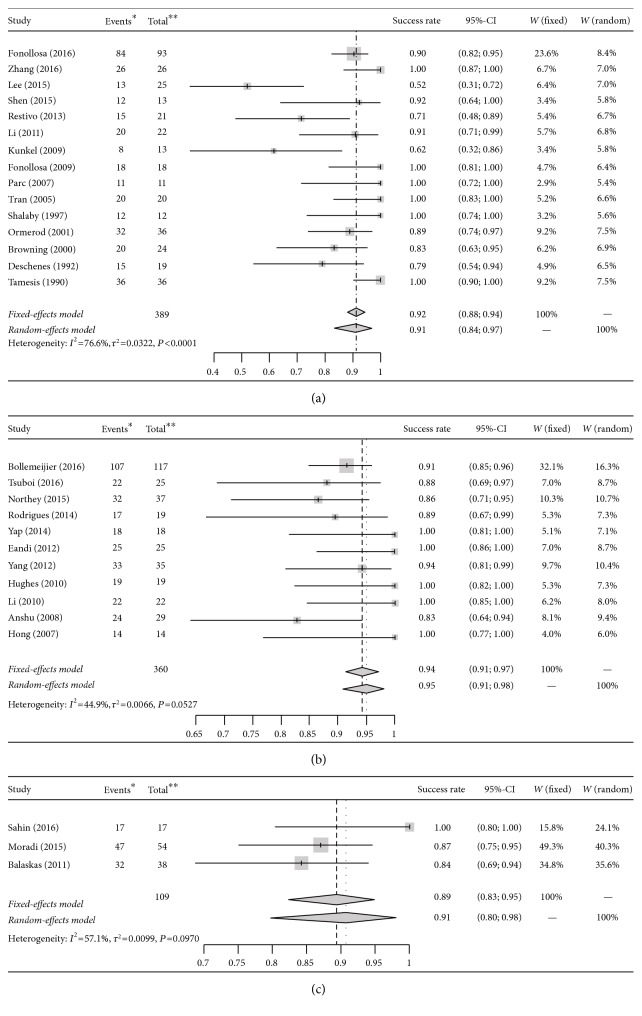
Forest plots of the success rates of antibacterial monotherapy (a); antibacterial agents and systemic corticosteroids (b); and antibacterial agents, systemic corticosteroids, and immunosuppressants (c). CI = confidence interval; W = weight; ^∗^number of eyes with final vision improved or maintained; ^∗∗^eyes included in studies.

Antibacterial agents were used as monotherapy in 286 patients in 15 studies [14, 17, 20, 23, 27, 30, 34, 35, 37, 39, 40, 42–45]. The mean age of the patients ranged from 37 to 58 years. The pooled success rate was 0.91 (95% CI 0.84–0.97) (Figure 2(a)). The heterogeneity of these studies was high (*P*_heterogeneity_ < 0.0001; *I*^2^ statistic = 76.6%).

### 3.6. Antibacterial Agents and Systemic Corticosteroids

Antibacterial agents were used in combination with systemic corticosteroids in some patients in 11 studies involving 245 patients [15, 19, 21, 24, 25, 28, 29, 32, 33, 36, 38]. The mean age of the patients ranged from 38 to 57.7 years. The pooled success rate was 0.95 (95% CI 0.91–0.98) (Figure 2(b)). Study heterogeneity was low (*P*_heterogeneity_ = 0.0527; *I*^2^ statistic = 44.9%).

### 3.7. Antibacterial Agents, Systemic Corticosteroids, and Immunosuppressants

Antibacterial agents were used in combination with systemic corticosteroids and immunosuppressants in some patients in 3 studies involving 73 patients [16, 22, 31]. The immunosuppressants included methotrexate, mycophenolate mofetil, cyclophosphamide, and cyclosporine. Further information on how many patients received each of these individual drugs was not available. The mean age of the patients ranged from 43.75 to 45 years. The pooled success rate was 0.91 (95% CI 0.80–0.98) (Figure 2(c)). Study heterogeneity was classified as moderate (*P*_heterogeneity_ = 0.0970; *I*^2^ statistic = 57.1%).

### 3.8. Recurrence, Complications, and Adverse Events

Recurrence of ocular inflammation was assessed in the follow-up period in 13 studies involving 210 patients [14, 15, 24, 27, 28, 31–33, 36, 37, 39, 40, 43]. The mean follow-up time ranged from 1 to 29.4 months in these studies. The pooled estimated recurrence rate was 10.7%.

Eight studies [17, 21, 22, 24, 27, 28, 30, 36] involving 190 patients (318 eyes) reported ocular complications that included cataract (*n* = 41), ocular hypertension (*n* = 15), posterior synechiae (*n* = 15), chorioretinal scarring (*n* = 12), epiretinal membrane (*n* = 12), macular edema (*n* = 10), optic disc atrophy (*n* = 10), and retinal detachment (*n* = 8) (Table 5).

Systemic adverse events (Jarisch-Herxheimer reaction) occurred in 3 patients enrolled in 3 studies involving 138 participants.

### 3.9. Subgroup Analysis

The outcomes of antibacterial monotherapy were assessed in subgroups of patients divided according to the study characteristics, and the results are presented in Table 6. Factors including the year of publication, the number of the eyes involved, HIV coinfection positivity, follow-up duration, and the geographical location of the patients were examined as potential sources of heterogeneity. However, we found no correlation of these study characteristics with the efficacy of antibacterial monotherapy. Nevertheless, there was substantial heterogeneity in the subgroups so *P* values for between-subgroup comparisons could not be calculated. Subgroup analyses were not possible for the other treatment regimens owing to the limited data on the drugs used.

### 3.10. Sensitivity and Publication Bias

**Figure 3:**
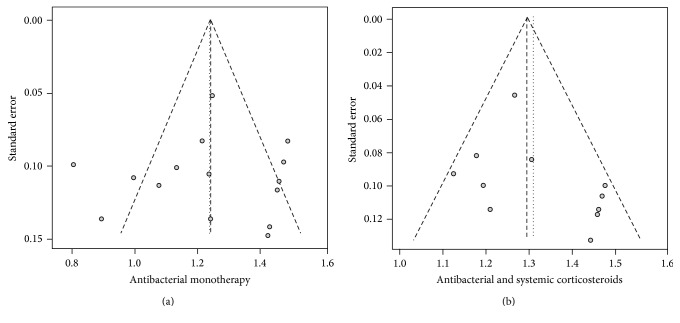
Funnel plots showing the standard error of standardized differences in the mean success rates for antibacterial monotherapy (a) and antibacterial agents and systemic corticosteroids (b). *x*-axes: Freeman-Tukey double arcsine transformed proportion.

In the sensitivity analysis (Figure 2), the results were similar using both fixed-effects and random-effects models. The pooled success rates using the fixed-effects and random-effects models were as follows: 0.92 (95% CI 0.88–0.94) and 0.91 (95% CI 0.84–0.97), respectively, for antibacterial monotherapy; 0.94 (95% CI 0.91–0.97) and 0.95 (95% CI 0.91–0.98), respectively, for antibacterial agents combined with systemic corticosteroids; and 0.89 (95% CI 0.83–0.95) and 0.91 (95% CI 0.80–0.98), respectively, for antibacterial agents combined with systemic corticosteroids and immunosuppressants. The funnel plots revealed no asymmetry (Figure 3). No evidence of publication bias was revealed using Begg's and Egger's regression tests, with *P* values of 0.85 for antibacterial monotherapy and 0.22 for antibacterial agents combined with systemic corticosteroids. The regression test was not done for triple therapy because of the small number of studies.

## 4. Discussion

This was the first systematic review to examine the efficacies of treatments for syphilitic uveitis. Most studies focusing on the treatment of syphilitic uveitis were mainly conducted retrospectively, being cohort or case series. Thus, the strength of the results of our systematic review may be low. However, because there are very few randomized controlled trials (RCTs) in this setting, our systematic review of non-RCTs provides valuable information on the clinical features and management of syphilitic uveitis. Our review comprised 670 patients across 32 studies, which were performed in multiple clinical centers in different countries with various ethnicities. Therefore, the results should be representative of the broader population of patients with syphilitic uveitis.

The demographic characteristics of the studies included in our review are consistent with current literature on syphilitic uveitis. In particular, syphilitic uveitis is most common in men aged 37–58 years, especially in men who have sex with men. HIV coinfection was also common, supporting the screening for HIV in patients with syphilitic uveitis.

For a long time, researchers have debated whether ocular syphilis is a subtype of neurosyphilis. The data collected in our review revealed that the optic disc (reported in 28/32 studies) might be the most commonly involved in patients with syphilitic uveitis, presenting as papillitis, optic neuritis, or neuroretinis, and panuveitis was reported in 24 of 32 studies, often in the presence of retinitis or optic neuritis. These findings are consistent with the idea that ocular syphilis is a manifestation of neurosyphilis in some patients. CSF samples were tested in many patients to detect neurosyphilis. Patients with a CSF WBC count > 10 cells/ml, protein level > 50 mg/dl, or reactive VDRL were considered to have neurosyphilis. The cumulative VDRL positivity rate of 34.8% suggests that one in three patients with syphilitic uveitis might have neurosyphilis. However, because CSF tests with negative VDRL results do not necessarily rule out neurosyphilis [48], many clinicians agree that treatment of syphilitic uveitis should follow the treatment of neurosyphilis [49, 50].

In our analysis, 91% (95% CI 84 to 97) of treated patients experienced improved or maintained visual function following antibacterial monotherapy. Our analysis did not reveal any additional benefit of coadministering systemic corticosteroids or immunosupressants with antibacterial agents. In practice, clinicians often prescribe systematic corticosteroids or immunomodulary agents in combination with antibacterial agents to patients with severe ocular inflammation or chronic macular edema [51, 52]. However, there is no consensus regarding the treatment of these conditions, and a systematic review is underway to evaluate the effectiveness of treatments for uveitic macular edema [53]. Prospective multicenter RCTs are needed to provide definitive evidence on the use of systematic corticosteroids or immunosuppressants. A systematic review [54] of penicillin and non-penicillin regimens for syphilis included 11 RCTs, but the authors reported that the “evidence defining treatment for late syphilis or HIV-infected persons is limited.”

Predictive factors associated with final visual acuity are of clinical interest. Our subgroup analysis did not reveal any correlation of the efficacy of antibacterial monotherapy with relevant study characteristics. In fact, only 7/32 studies reported possible factors associated with final visual acuity. Factors associated with poor visual prognosis included the time between onset of uveitis and treatment (>12 weeks), longer duration of ocular symptoms (>28 days), presence of macular edema or long-standing optic neuropathy, coinfection with HIV, and poor initial visual acuity. Factors associated with higher success rates included the presence of vasculitis (as detected by fundus fluorescence angiography), anterior uveitis, or neurosyphilis.

The treatment outcomes for patients with syphilitic uveitis and HIV coinfection are of considerable interest. We performed subgroup analysis to examine the efficacy of antibacterial monotherapy for syphilitic uveitis in 15 studies, which included several studies involving patients with HIV coinfection. However, stratification did not reveal any correlations of the efficacy of antibacterial monotherapy with study characteristics, including HIV coinfection status. In fact, nine studies included subgroup analyses of HIV-positive and HIV-negative patients and the factors influencing final visual acuity were investigated in these studies. Although some studies revealed a higher incidence of panuveitis in HIV-positive patients, the data for eight of the nine studies included in our analysis were inadequate to determine whether specific subtypes of uveitis were more common in HIV-positive patients. In addition, in seven of these nine studies, the authors found no differences in visual prognosis between HIV-positive and HIV-negative patients. Tucker et al. [10] performed a systematic analysis of 101 HIV-positive patients using data published in case series and case reports. They reported that “ocular syphilis led to the HIV diagnosis in 52% of cases, posterior uveitis was significantly more common in individuals with CD4 cell count < 200 cells/mm^3^, and 97% of patients with visual impairment improved following intravenous penicillin or ceftriaxone.” In our analysis, the mean CD4 cell count ranged from 122 to 504 cells/mm^3^, supporting the recommendations that most patients with syphilitic uveitis and HIV coinfection should be treated according to the recommendations for HIV-negative patients (e.g., as immunocompetent patients) and should be monitored closely [1].

There were several reviews on syphilitic uveitis or ocular syphilis. Woolston et al. [49] recently summarized the epidemiology and spectrum of the ocular findings of patients with ocular syphilis and supported the concept that ocular syphilis should be treated like neurosyphilis. Davis [50] described the clinical features of ocular syphilis, including some relatively unique signs of syphilitic uveitis, such as preretinal opacities or acute syphilitic posterior placoid choroiditis. They stated that the “treatment for syphilitic uveitis always follows the treatment regimen used for neurosyphilis and requires an examination of the cerebrospinal fluid.” However, the outcomes of treatments were not analyzed in either review. Amaratunge et al. [55] reviewed cases of syphilitic uveitis reported between 1984 and 2008 in 41 articles and identified 143 patients with syphilitic uveitis, of which 65.0% had HIV coinfection. They reported that posterior uveitis was the most common phenotype (55.2%) and CSF abnormalities were more common in HIV-positive patients (76%); however, their results are not consistent with our results.

The good overall prognosis reported in prior studies suggests that further clinical trials of syphilitic uveitis are not urgently required. However, further studies may be valuable to establish the indications for corticosteroids or immunosuppressants. In addition, factors associated with final visual acuity and factors associated with HIV coinfection still needed to be investigated in suitably large studies. Because the global incidence of syphilitic uveitis is low, multicenter studies may be appropriate. Furthermore, understanding the pathogenesis of syphilis is compromised because *Treponema pallidum* is an exclusively human pathogen that cannot be grown in vitro. Moreover, very few studies have performed culture or polymerase chain reaction assays of intraocular fluid from patients with syphilitic uveitis [56]. In addition, some phenotypes of syphilitic uveitis were rare. For example, necrotizing retinitis was only reported in five studies included in our analysis, but it could be confused with acute retinal necrosis caused by herpes viruses. The analysis of aqueous humor in patients with necrotizing retinitis might be valuable in the differential diagnosis and to investigate the pathogenesis of this phenotype. Thus, future studies using aqueous humor samples from eyes with syphilitic uveitis may provide unique insight into direct spirochete infection and the consequent immune reactions. Such information might facilitate individualized treatment of syphilitic uveitis in patients with poor visual prognosis based on the current recommended therapies.

## 5. Conclusions

Our systematic review summarized the complex clinical features of syphilitic uveitis, and our meta-analysis of published studies supports the use of antibacterial agents for treating syphilitic uveitis, regardless of HIV coinfection. However, we found no additional benefit of coadministering systemic corticosteroids and immunosuppressants with antibacterial agents relative to antibacterial monotherapy.

## Figures and Tables

**Table 1:** Study characteristics and patient demographics.

Study	Study design	Study period	Geographical location	Number of patients	Number of eyes	Number of dropouts or loss of follow-up	Age (years), range	Males (%)	Ethnicity (%)
Bollemeijier (2016)	Retrospective	1984–2013	Netherlands	85	139	10	47 (27–73)	82.4	Caucasian (78.8), Surinam black (8.2), African American (2.3), Asian (8.2), Surinam Indian (2.3)
Dai (2016)	Retrospective	2011-2012	China	25	41	Unknown	53 (33–70)	72	Chinese (100.0)
Fonollosa (2016)	Retrospective	2000–2012	Spain	50	93	0	41 (19–76)	61	—
Sahin (2016)	Retrospective	2012–2014	Turkey	12	17	0	43.75 (8–67)	58.3	Caucasian (100.0)
Tsuboi (2016)	Retrospective	1997–2015	Japan	20	30	4	41 (32.5–46.5)	100	Japanese (100.0)
Zhang (2016)	Retrospective	2012–2015	China	15	26	0	50 (35–68)	60	Chinese (100.0)
Lee (2015)	Retrospective	2008–2014	USA	16	29	2	48.5 (20–63)	100	Black (25.0), Hispanic (56.2), white (12.5), others (6.2)
Moradi (2015)	Retrospective	1984–2014	USA	35	61	4	45 (24–80)	74.3	African American (60.0), Caucasian (40.0)
Northey (2015)	Retrospective	2007–2012	Australia	25	37	50% lost after 3 months	41 (24–81)	92	—
Shen (2015)	Retrospective	2009–2014	China	13	21	4	50.3 ± 5.9 (37–61)	53.8	Chinese (100.0)
Mathew (2014)	Prospective	2009–2011	UK	41	63	0	47.5 (20.6–75.1)	90.2	Caucasian (90.2), Afro Caribbean (7.1); Arab (2.4)
Rodrigues (2014)	Retrospective	2012 (Mar. to Oct.)	Brazil	12	19	Unknown	38.5 (28–55)	91.6	—
Yap (2014)	Retrospective	2004–2009	Singapore	12	18	2 only at 1 month	49.5 (24–84)	91.7	Chinese (75.0), Malays (16.7), Indian (8.3)
Restivo (2013)	Retrospective	2004–2010	Italy	14	21	0	46.2 ± 10.4 (26–66)	92.9	—
Eandi (2012)	Retrospective	—	USA, Europe	16	25	Unknown	40 (28–57)	87.5	Non-Latino white (93.8), black (6.3)
Yang (2012)	Retrospective	2004–2011	China	19	35	0	41.8 (19–70)	57.9	Asian (100.0)
Balaskas (2011)	Retrospective	1999–2009	Switzerland	26	42	3	45 (33–80)	69.2	Caucasian (80.8), African (15.4), Asian (3.8)
Li (2011)	Retrospective	1991–2009	USA	13	24	1	40 (30–67)	100	—
Hughes (2010)	Retrospective	2006–2009	Australia	13	19	0	41.7 (29–29)	92.3	—
Li (2010)	Retrospective	92% after 2000	Not mentioned	13	22	2-3 probably lost	38 (26–55)	100	—
Kunkel (2009)	Retrospective	1998–2006	Germany	24	41	11	42.4 ± 2.5 (*n* = 13); 41.6 ± 3.0 (*n* = 11)	91.7	—
Fonollosa (2009)	Retrospective	2005–2007	Spain	12	18	0	47 (26–76)	83.3	—
Anshu (2008)	Retrospective	1995–2006	Singapore	22	29	Unknown	52.7 (18–78)	77.3	—
Hong (2007)	Retrospective	1992–2004	Taiwan	8	14	0	57.7 (32–82)	87.5	—
Parc (2007)	Retrospective	2001–2004	France	10	11	6/10 lost after 3 months	41.2 ± 9.9 (28–59)	100	—
Tran (2005)	Retrospective	2001–2003	France	12	20	3	40 (28–56)	100	—
Shalaby (1997)	Retrospective	1983–1995	USA	13	23	5	37 (?–?)	92.3	—
Ormerod (2001)	Retrospective	1990–1993	USA	21	40	2	51 (29–72)	61.9	Black (76.2), no data (23.8)
Browning (2000)	Retrospective	1986–1999	USA	14	24	0	42 (28–69)	71.4	African American (78.6), Caucasian (21.4)
Villanueva (2000)	Retrospective	1993–1996	USA	20	Not mentioned	6	58 ± 14 (29–70)	40	Black (100.0)
Deschenes (1992)	Retrospective	1986–1990	Canada	14	24	Unknown	Mean: 50 for men, 63 for women	64.3	—
Tamesis (1990)	Retrospective	1983–1989	USA	25	36	Unknown	Mean: 47 for men, 57 for women	40	—

UK: United Kingdom; USA: United States of America.

**Table 2:** Clinical characteristics of patients with syphilitic uveitis.

Study	Bilaterality (%)	AU (%)	IU (%)	PU (%)	PAU (%)	ASPPC (%)	Retinitis, retinal vasculitis, neuroretinitis	Papillitis, optic neuritis	HIV-positive (%)	CSF VDRL positive (%)
Bollemeijier (2016)	63.5	15.8	1.4	30.2	47.5	—	86.0	74.0	35.9	38.7
Dai (2016)	20.0	—	—	—	—	—	22.0	14.6	0.0	36.0
Fonollosa (2016)	86.0	14.0	0.0	52.0	34.0	8.6	23.7	33.3	34.0	15.0
Sahin (2016)	41.7	29.4	11.8	29.4	11.8	—	—	5.9	0.0	—
Tsuboi (2016)	50.0	10.0	6.7	50.0	20.0	6.7	—	53.3	100.0	—
Zhang (2016)	73.3	—	—	—	—	3.0	17.0	9.0	13.3	—
Lee (2015)	81.3	13.8	—	27.6	44.8	—	—	13.8	62.5	20.0
Moradi (2015)	74.3	36.1	23.0	8.2	45.9	3.3	—	—	54.3	47.1
Northey (2015)	52.0	32.4	—	67.6	2.7	2.7	27.0	27.0	32.0	42.9
Shen (2015)	61.5	4.8	—	—	9.5	—	52.4	14.3	7.7	—
Mathew (2014)	56.0	9.5	1.6	12.7	41.3	—	—	22.2	31.7	—
Rodrigues (2014)	58.3	5.3	—	21.1	57.9	—	5.3	10.5	66.7	22.2
Yap (2014)	50.0	33.3	5.6	27.8	33.3	—	44.4	33.3	25.0	44.4
Restivo (2013)	50.0	—	9.5	28.6	50.0	—	—	—	42.9	42.9
Eandi (2012)										
Yang (2012)	84.2	—	—	85.7	—	5.7	94.3	28.6	21.0	—
Balaskas (2011)	61.5	0.0	—	50.0	45.2	—	69.0	31.0	7.7	5.5
Hughes (2010)	46.2	10.5	10.5	36.8	47.4	—	73.7	—	46.1	60.0
Li (2010)	69.2	22.7	—	23.1	30.8	—	—	27.3	100.0	50.0
Kunkel (2009)	71.0	17.1	—	41.7	9.8	—	—	26.8	45.8	29.1
Fonollosa (2009)	50.0	—	—	5.6	94.4	—	27.8	27.8	75.0	75.0
Anshu (2008)	31.8	75.9	10.3	13.8	27.6	3.4	27.6	27.6	0.0	50.0
Hong (2007)	75.0	—	—	7.1	78.6	—	14.3	28.6	0.0	20.0
Parc (2007)	10.0	—	—	100.0	—	—	36.4	72.7	80.0	22.2
Tran (2005)	67.0	10.0	—	—	20.0	30.0	35.0	25.0	100.0	—
Shalaby (1997)	83.0	30.8	23.1	—	38.5	—	—	7.7	100.0	63.6
Li (2011)	85.0	—	—	41.7	58.3	—	66.7	41.7	83.0	25.0
Ormerod (2001)	90.4	—	—	—	5.0	2.0	17.5	2.5	33.3	47.4
Browning (2000)	71.4	—	—	—	—	—	54.2	12.5	36.0	22.2
Villanueva (2000)	—	—	—	—	15.0	—	10.0	—	33.3	20.0
Deschenes (1992)	71.4	85.7	—	>0	>0	—	28.6	14.3	66.7	—
Tamesis (1990)	44.4	29.4	5.9	17.6	47.1	—	11.8	17.6	8.0	50.0

ASPPC: acute syphilitic posterior placoid choroiditis; AU: anterior uveitis; CSF: cerebrospinal fluid; HIV: human immunodeficiency virus; IU: intermediate uveitis; PAU: panuveitis; PU: posterior uveitis; VDRL: venereal disease research laboratory test.

**Table 3:** Clinical phenotypes of syphilitic uveitis.

Phenotype	Number of studies (%)^∗^
Papillitis, optic neuritis, or neuroretinitis	28 (87.5)
Panuveitis	24 (75.0)
Retinitis, retinal vasculitis	23 (71.9)
Anterior uveitis	21 (65.6)
Posterior uveitis	20 (62.5)
ASPPC	10 (31.3)
Choroiditis or chorioretinitis	8 (25.0)
Intermediate uveitis	11 (34.4)
Macular edema	7 (21.9)
IOP increased	6 (18.8)
Serous retinal detachment	6 (18.8)
Necrotizing retinitis	5 (15.6)

ASPPC: acute syphilitic posterior placoid choroiditis; IOP: intraocular pressure; ^∗^number of studies that included patients with the specified clinical phenotype (of 32 studies included in this review).

**Table 4:** Treatments and outcomes.

Study	Intervention			Mean follow-up time (months)	Outcome	
	Antibacterial agents	Steroids	Immunosuppressant		Outcome measures	Improvement or maintenance in visual acuity (%)
Bollemeijier (2016)	Benzyl PNC 0.15 MU/kg/d IV (14 d), or procaine PNC 1.2–2.4 MU IV (10–17 d), or doxycycline 200 mg po bid (28 d), or ceftriaxone IV 2 g/d (14 d)	±systemic, ±topical, ±subconjunctival	—	6	Improvement in visual acuity, factors associated with final visual acuity	91.5
Dai (2016)	—	—	—	—	—	—
Fonollosa (2016)	74%: penicillin IV or IM, or combined. Others: oral doxycycline and ceftriaxone either IM or IV	—	—	14	Improvement in visual acuity, ocular complications	90.0
Sahin (2016)	PCN 24 MU/day IV (10 d)	±systemic	±	>6	Improvement in visual acuity and inflammation	100.0
Tsuboi (2016)	75% benzyl PNC 24 MU/d IV, amoxicillin po plus probenecid before or after	±systemic	—	21	Improvement in visual acuity and inflammation	100.0
Zhang (2016)	All PNC G 18–24 MU/d IV (2 w), or ceftriaxone 2 g/d IV (2 w)	—	—	10.1	Improvement in visual acuity and inflammation	88.0
Lee (2015)	Benzyl PNC 18–24 MU/d IV (1014 d), or IV ceftriaxone (14 d), doxycycline po (21 d)	—	—	1–96	Improvement in visual acuity	100.0
Moradi (2015)	PNC IV/IM, or ceftriaxion	±systemic	±	9	Incidence of visual loss, ocular complications	52.0
Northey (2015)	Benzyl PNC IV (14 d), benzathine PNC IM, doxycycline po	±systemic, ±topical	—	6	Improvement in visual acuity and inflammation	87.0
Shen (2015)	Benzathine IM or ceftriaxone IV or doxycycline po	—	—	4.1	Improvement in visual acuity	92.3
Mathew (2014)	—	—	—	>6	Improvement in visual acuity	93.7
Rodrigues (2014)	Crystalline PNC 20 MU/d IV (14 d)	±systemic	—	—	Improvement in visual acuity	89.5
Yap (2014)	PNC IV (10–14 d), or procaine PNC IM (10 d) + benzathine PNC G IM, or benzathine penicillin G IM	±systemic, ±topical	—	>3 (10/12 pt.)	Improvement in visual acuity and inflammation	100.0
Restivo (2013)	PNC 24 MU/d (14 d)	Topical	—	29.4	Improvement in visual acuity and inflammation	71.4
Eandi (2012)	PNC 24 MU/d (14 d)	Systemic	—	2.9	Improvement in visual acuity and inflammation	100.0
Yang (2012)	PNC 18–24 MU/d IV (14–20 d), or doxycycline po	Systemic (taper)	—	9.3	Improvement in visual acuity and inflammation	94.3
Balaskas (2011)	PNC IV, or ceftriaxone	±systemic	±	10	Improvement in visual acuity	84.2
Hughes (2010)	PNC IV (10–14 d)	±systemic	—	—	Improvement in visual acuity and inflammation	100.0
Li (2010)	PNC IV (2 w) + PNC G benzathine IM (1–3 w), or doxycycline po (4 w)	±systemic (taper)	—	—	Improvement in visual acuity	100.0
Kunkel (2009)	PNC G IV or ceftriaxone (≥10 d)	—	—	—	Improvement in visual acuity	61.5
Fonollosa (2009)	PNC G IV 24 MU/d (14 d)	±intravitreal	—	19.8	Improvement in visual acuity	100.0
Anshu (2008)	Crystalline PNC G (18–24 MU/d) or procaine PNC (2–4 MU/d IM) with probenecid (10–14 d), or ceftriazone 2 g/d (10–14 d)	±systemic, ±topical	—	—	Improvement in visual acuity	82.8
Hong (2007)	Crystal PNC G 12–18 MU/d (6–14 d) + PNC G 2.4 MU/w IM (3-4 w), or PNC IV (10–14 d), or tetracycline 500 mg po qd (26 d)	±systemic prior to diagnosis	—	4.3	Improvement in visual acuity and inflammation	100.0
Parc (2007)	PNC G IV, or cetriaxone IV, or benzathine-benzyl PNC IM	—	—	>1 (all pt.); 5 (4 pt.)	Improvement in visual acuity and inflammation	100.0
Tran (2005)	PNC 24 MU/day IV (≥14 d), or ceftriaxone 2 g/day IV (21 d)	—	—	7	Improvement in visual acuity and inflammation	100.0
Shalaby (1997)	PNC 12–24 MU/d IV (10–14 d), or ceftriaxone 2 g/d IV (10 d)	Topical	—	>3 (8/13 pt.)	Improvement in visual acuity and inflammation	100.0
Li (2011)	PNC IV (≥10 d), or PNC IM, or ceftriaxone IM + PCN IV bolus, or tetracycline po	—	—	31	Improvement in visual acuity and inflammation	90.9
Ormerod (2001)	Benzyl PNC IV (10–14 d), or a combination of PNC IV + benzathine PNC IM, or benzathine PNC IM alone	—	—	5	Improvement in visual acuity and inflammation	88.9
Browning (2000)	PNC IV (10 d), or benzathine PNC IM + probenecid po	—	—	6	Improvement in visual acuity and inflammation	83.3
Villanueva (2000)	Crystalline PNC G 12–24 MU/d IV	±topical	—	12.9	Improvement in ocular lesions	—
Deschenes (1992)	PNC 18–24 MU/d IV (10–14 d) + PNC 2.4 MU/w IM, or PNC IM (2-3 w), or tetracycline	—	—	—	Improvement in visual acuity	78.9
Tamesis (1990)	PNC, no details	±topical	—	10	Improvement in visual acuity and inflammation	100.0

ASPPC: acute syphilitic posterior placoid choroiditis; bid: twice daily; CSF: cerebrospinal fluid; d: days; IM: intramuscular; IV: intravenous; MU: million unit; PNC: penicillin; po: per os (oral).

**Table 5:** Ocular complications and systemic adverse events (318 eyes).

Complications	Number of events (proportion, %)	*N*/*N*^a^ (%)
Ocular complications		
Cataract	41 (29.5)	12.9
Ocular HTN	15 (10.8)	4.7
PS	15 (10.8)	4.7
Chorioretinal scarring	12 (8.6)	3.8
ERM	12 (8.6)	3.8
ME	10 (7.2)	3.1
Optic disc atrophy	10 (7.2)	3.1
RD	8 (5.8)	2.5
PVR	5 (3.6)	1.6
Phthisis bulbi	4 (2.9)	1.3
Others	7 (5.0)	2.2
Total	139 (100)	43.7
Systemic adverse events		
Jarisch-Herxheimer reaction	3	2.2 (of 136 patients)

ERM: epiretinal membrane; HTN: hypertension; ME: macular edema; PS: posterior synechiae; PVR: proliferative vitreoretinopathy; RD: retinal detachment; ^a^percentage of eyes with ocular complications in a total of 318 eyes included in eight studies that reported complications.

**Table 6:** Subgroup analysis of efficacy of antibacterial monotherapy of syphilitic uveitis.

Subgroup	Number of studies	Pooled success rate (95% CI)	*I* ^2^ (%)	*P*_heterogeneity_
Publication year				
>2009	6	0.86 (0.71–0.97)	82.4	<0.0001
≤2009	9	0.94 (0.85–1.00)	70.6	0.0007
Number of eyes				
<25	9	0.93 (0.85–0.99)	57.9	0.0149
≥25	6	0.88 (0.72–0.98)	87.7	<0.0001
Geographical location				
North America	7	0.88 (0.74–0.98)	81.5	<0.0001
Europe	6	0.92 (0.79–0.99)	75.7	0.0010
China	2	0.98 (0.85–1.00)	48.2	0.1648
HIV positivity (%)				
≥50	7	0.93 (0.78–1.00)	81.3	<0.0001
<50	8	0.90 (0.80–0.97)	74.6	0.0003
Follow-up duration (months)				
≥12	4	0.90 (0.79–0.98)	64.9	0.0151
<12	7	0.97 (0.91–1.00)	55.8	0.0348
Unclear	4	0.76 (0.50–0.95)	78.9	0.0026

CI: confidence interval; HIV: human immunodeficiency virus.
